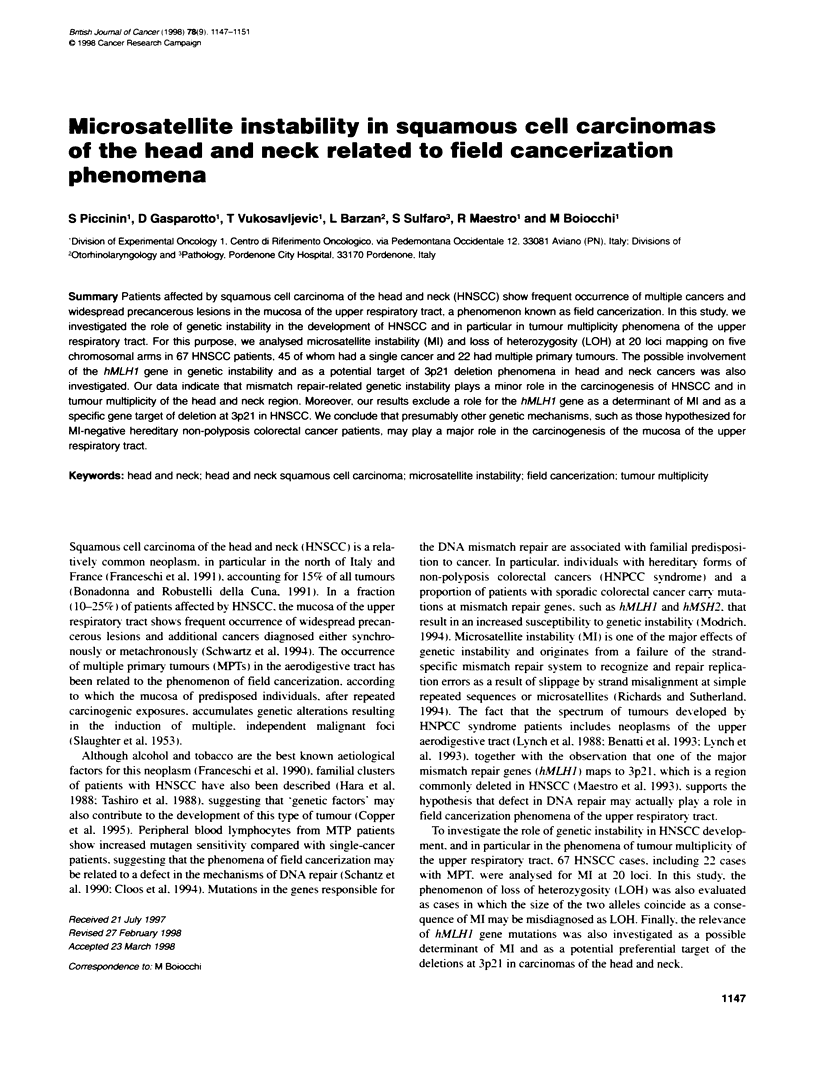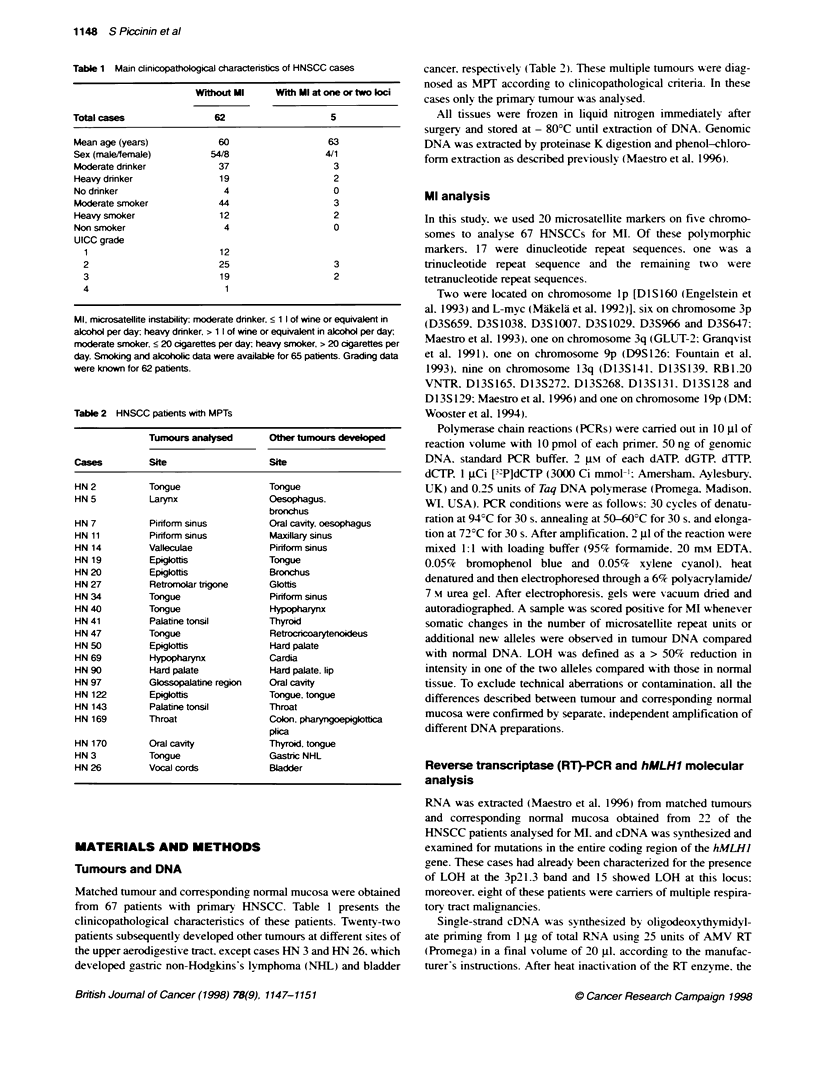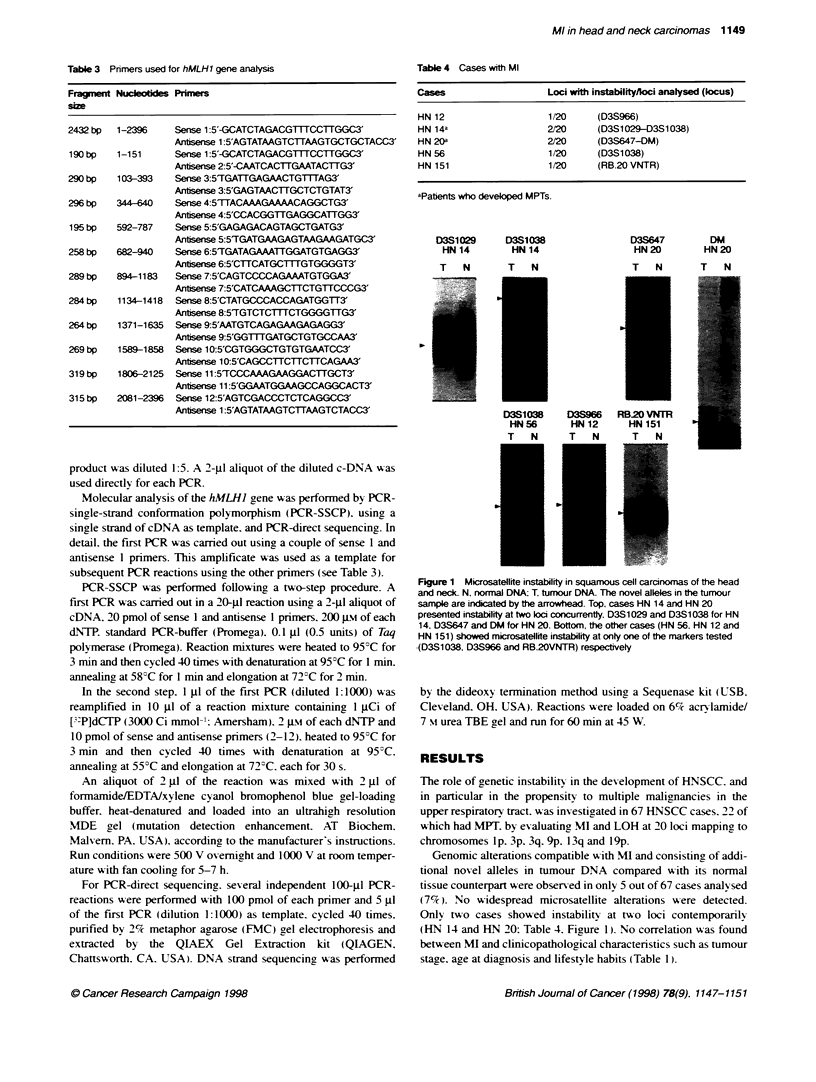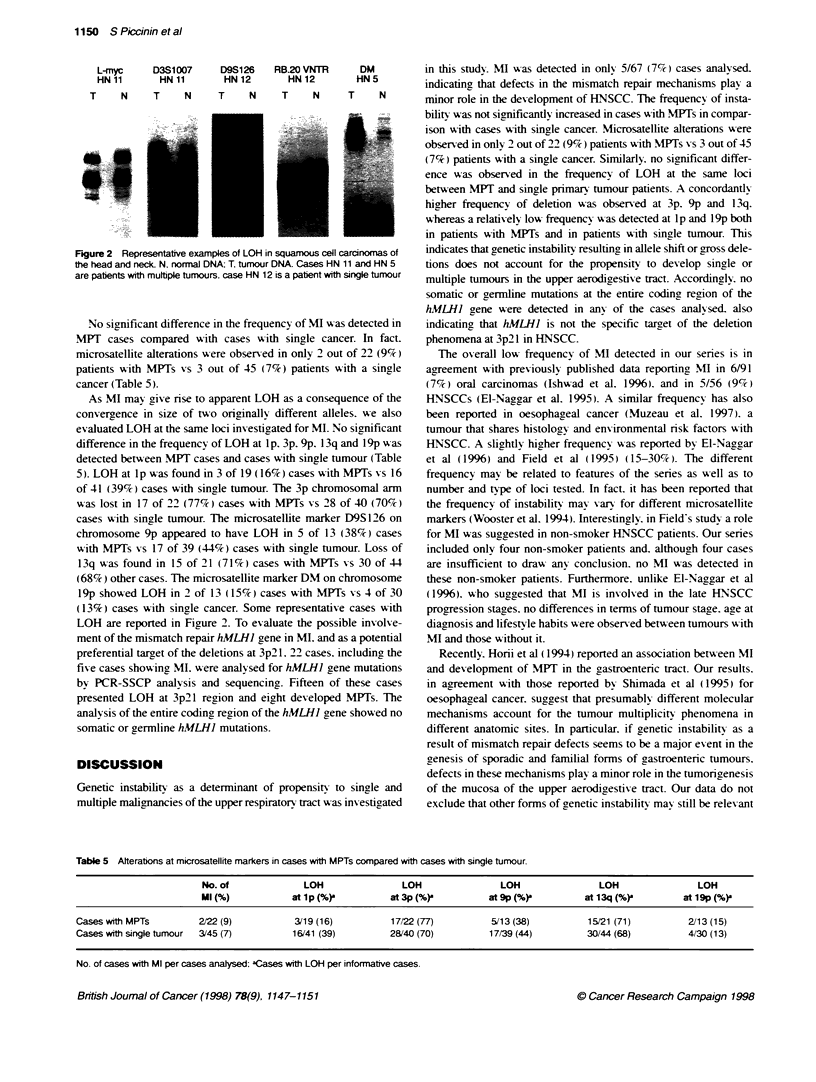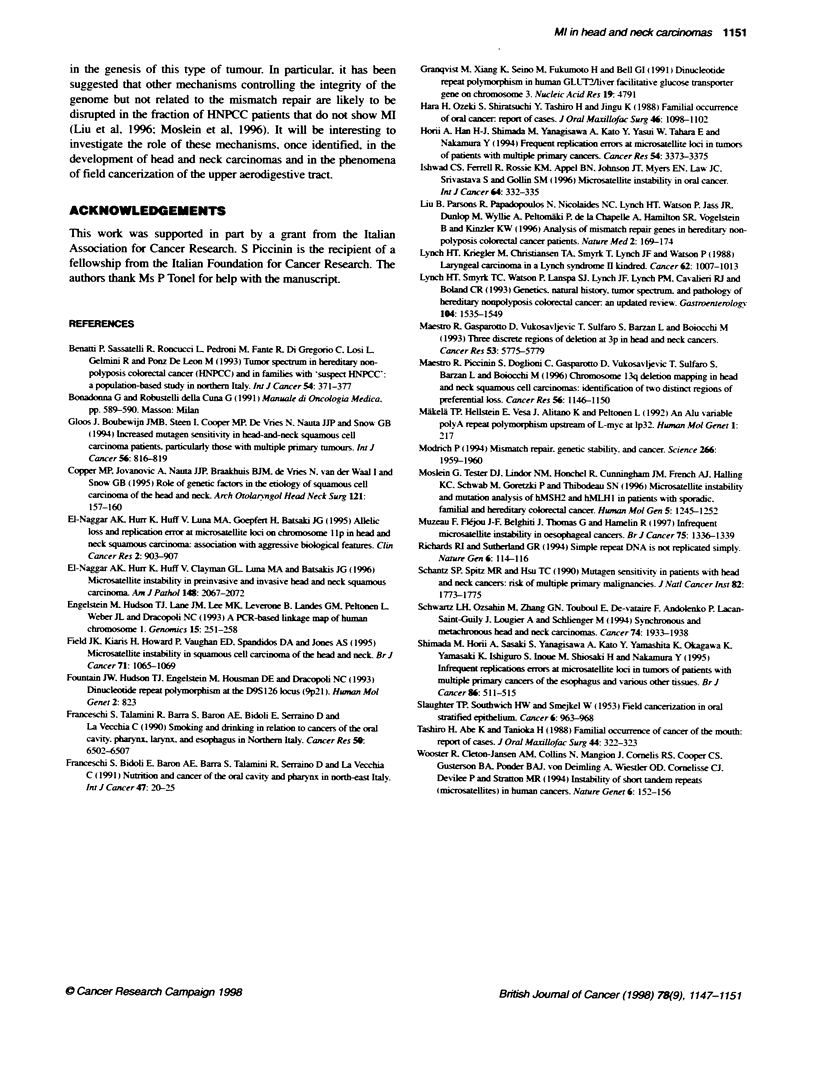# Microsatellite instability in squamous cell carcinomas of the head and neck related to field cancerization phenomena.

**DOI:** 10.1038/bjc.1998.644

**Published:** 1998-11

**Authors:** S. Piccinin, D. Gasparotto, T. Vukosavljevic, L. Barzan, S. Sulfaro, R. Maestro, M. Boiocchi

**Affiliations:** Division of Experimental Oncology 1, Centro di Riferimento Oncologico, Aviano (PN), Italy.

## Abstract

**Images:**


British Journal of Cancer (1998) 78(9). 1147–1151
© 1998 Cancer Research Campaign

### Microsatellite instability in squamous cell carcinomas of the head and neck related to field cancerization phenomena

S Piccinin[1], D Gasparotto[1], T Vukosavljevic[1], L Barzan[2], S Sulfaro[3], R Maestro[1] and M Boiocchi[1]

[1]Division of Experimental Oncology 1. Centro di Riferimento Oncologico. via Pedemontana Occidentale 12. 33081 Aviano (PN). Italy: Divisions of [2]Otorhinolaryngology and [3]Pathology. Pordenone City Hospital. 33170 Pordenone. Italy

**Summary** Patients affected by squamous cell carcinoma of the head and neck (HNSCC) show frequent occurrence of multiple cancers and widespread precancerous lesions in the mucosa of the upper respiratory tract, a phenomenon known as field cancerization. In this study, we investigated the role of genetic instability in the development of HNSCC and in particular in tumour multiplicity phenomena of the upper respiratory tract. For this purpose, we analysed microsatellite instability (MI) and loss of heterozygosity (LOH) at 20 loci mapping on five chromosomal arms in 67 HNSCC patients, 45 of whom had a single cancer and 22 had multiple primary tumours. The possible involvement of the *hMLH1* gene in genetic instability and as a potential target of 3p21 deletion phenomena in head and neck cancers was also investigated. Our data indicate that mismatch repair-related genetic instability plays a minor role in the carcinogenesis of HNSCC and in tumour multiplicity of the head and neck region. Moreover, our results exclude a role for the *hMLH1* gene as a determinant of MI and as a specific gene target of deletion at 3p21 in HNSCC. We conclude that presumably other genetic mechanisms, such as those hypothesized for MI-negative hereditary non-polyposis colorectal cancer patients, may play a major role in the carcinogenesis of the mucosa of the upper respiratory tract.

**Keywords:** head and neck; head and neck squamous cell carcinoma; microsatellite instability; field cancerization; tumour multiplicity

Squamous cell carcinoma of the head and neck (HNSCC) is a relatively common neoplasm. in particular in the north of Italy and France (Franceschi et al. 1991). accounting for 15% of all tumours (Bonadonna and Robustelli della Cuna. 1991). In a fraction (10–25%) of patients affected by HNSCC. the mucosa of the upper respiratory tract shows frequent occurrence of widespread precancerous lesions and additional cancers diagnosed either synchronously or metachronously (Schwartz et al. 1994). The occurrence of multiple primary tumours (MPTs) in the aerodigestive tract has been related to the phenomenon of field cancerization. according to which the mucosa of predisposed individuals. after repeated carcinogenic exposures. accumulates genetic alterations resulting in the induction of multiple. independent malignant foci (Slaughter et al. 1953).

Although alcohol and tobacco are the best known aetiological factors for this neoplasm (Franceschi et al. 1990). familial clusters of patients with HNSCC have also been described (Hara et al. 1988: Tashiro et al. 1988). suggesting that 'genetic factors' may also contribute to the development of this type of tumour (Copper et al. 1995). Peripheral blood lymphocytes from MTP patients show increased mutagen sensitivity compared with single-cancer patients. suggesting that the phenomena of field cancerization may be related to a defect in the mechanisms of DNA repair (Schantz et al. 1990: Cloos et al. 1994). Mutations in the genes responsible for the DNA mismatch repair are associated with familial predisposition to cancer. In particular. individuals with hereditary forms of non-polyposis colorectal cancers (HNPCC syndrome) and a proportion of patients with sporadic colorectal cancer carry mutations at mismatch repair genes. such as *hMLH1* and *hMSH2*. that result in an increased susceptibility to genetic instability (Modrich. 1994). Microsatellite instability (MI) is one of the major effects of genetic instability and originates from a failure of the strand-specific mismatch repair system to recognize and repair replication errors as a result of slippage by strand misalignment at simple repeated sequences or microsatellites (Richards and Sutherland. 1994). The fact that the spectrum of tumours developed by HNPCC syndrome patients includes neoplasms of the upper aerodigestive tract (Lynch et al. 1988: Benatti et al. 1993: Lynch et al. 1993). together with the observation that one of the major mismatch repair genes (*hMLH1*) maps to 3p21. which is a region commonly deleted in HNSCC (Maestro et al. 1993). supports the hypothesis that defect in DNA repair may actually play a role in field cancerization phenomena of the upper respiratory tract.

To investigate the role of genetic instability in HNSCC development. and in particular in the phenomena of tumour multiplicity of the upper respiratory tract. 67 HNSCC cases. including 22 cases with MPT. were analysed for MI at 20 loci. In this study. the phenomenon of loss of heterozygosity (LOH) was also evaluated as cases in which the size of the two alleles coincide as a consequence of MI may be misdiagnosed as LOH. Finally. the relevance of *hMLH1* gene mutations was also investigated as a possible determinant of MI and as a potential preferential target of the deletions at 3p21 in carcinomas of the head and neck.

*Received 21 July 1997*
*Revised 27 February 1998*
*Accepted 23 March 1998*

*Correspondence to:* M Boiocchi

Table 1 Main clinicopathological characteristics of HNSCC cases

| | Without MI | With MI at one or two loci |
|---|---|---|
| **Total cases** | **62** | **5** |
| Mean age (years) | 60 | 63 |
| Sex (male/female) | 54/8 | 4/1 |
| Moderate drinker | 37 | 3 |
| Heavy drinker | 19 | 2 |
| No drinker | 4 | 0 |
| Moderate smoker | 44 | 3 |
| Heavy smoker | 12 | 2 |
| Non smoker | 4 | 0 |
| UICC grade | | |
| 1 | 12 | |
| 2 | 25 | 3 |
| 3 | 19 | 2 |
| 4 | 1 | |

MI, microsatellite instability; moderate drinker, ≤ 1 l of wine or equivalent in alcohol per day; heavy drinker, > 1 l of wine or equivalent in alcohol per day; moderate smoker, ≤ 20 cigarettes per day; heavy smoker, > 20 cigarettes per day. Smoking and alcoholic data were available for 65 patients. Grading data were known for 62 patients.

Table 2 HNSCC patients with MPTs

| | Tumours analysed | Other tumours developed |
|---|---|---|
| **Cases** | **Site** | **Site** |
| HN 2 | Tongue | Tongue |
| HN 5 | Larynx | Oesophagus, bronchus |
| HN 7 | Piriform sinus | Oral cavity, oesophagus |
| HN 11 | Piriform sinus | Maxillary sinus |
| HN 14 | Valleculae | Piriform sinus |
| HN 19 | Epiglottis | Tongue |
| HN 20 | Epiglottis | Bronchus |
| HN 27 | Retromolar trigone | Glottis |
| HN 34 | Tongue | Piriform sinus |
| HN 40 | Tongue | Hypopharynx |
| HN 41 | Palatine tonsil | Thyroid |
| HN 47 | Tongue | Retrocricoarytenoideus |
| HN 50 | Epiglottis | Hard palate |
| HN 69 | Hypopharynx | Cardia |
| HN 90 | Hard palate | Hard palate, lip |
| HN 97 | Glossopalatine region | Oral cavity |
| HN 122 | Epiglottis | Tongue, tongue |
| HN 143 | Palatine tonsil | Throat |
| HN 169 | Throat | Colon, pharyngoepiglottica plica |
| HN 170 | Oral cavity | Thyroid, tongue |
| HN 3 | Tongue | Gastric NHL |
| HN 26 | Vocal cords | Bladder |

### MATERIALS AND METHODS

### Tumours and DNA

Matched tumour and corresponding normal mucosa were obtained from 67 patients with primary HNSCC. Table 1 presents the clinicopathological characteristics of these patients. Twenty-two patients subsequently developed other tumours at different sites of the upper aerodigestive tract, except cases HN 3 and HN 26, which developed gastric non-Hodgkins's lymphoma (NHL) and bladder cancer, respectively (Table 2). These multiple tumours were diagnosed as MPT according to clinicopathological criteria. In these cases only the primary tumour was analysed.

All tissues were frozen in liquid nitrogen immediately after surgery and stored at – 80°C until extraction of DNA. Genomic DNA was extracted by proteinase K digestion and phenol–chloroform extraction as described previously (Maestro et al. 1996).

### MI analysis

In this study, we used 20 microsatellite markers on five chromosomes to analyse 67 HNSCCs for MI. Of these polymorphic markers, 17 were dinucleotide repeat sequences, one was a trinucleotide repeat sequence and the remaining two were tetranucleotide repeat sequences.

Two were located on chromosome 1p [D1S160 (Engelstein et al. 1993) and L-myc (Mäkelä et al. 1992)], six on chromosome 3p (D3S659, D3S1038, D3S1007, D3S1029, D3S966 and D3S647; Maestro et al. 1993), one on chromosome 3q (GLUT-2; Granqvist et al. 1991), one on chromosome 9p (D9S126; Fountain et al. 1993), nine on chromosome 13q (D13S141, D13S139, RB1.20 VNTR, D13S165, D13S272, D13S268, D13S131, D13S128 and D13S129; Maestro et al. 1996) and one on chromosome 19p (DM; Wooster et al. 1994).

Polymerase chain reactions (PCRs) were carried out in 10 µl of reaction volume with 10 pmol of each primer, 50 ng of genomic DNA, standard PCR buffer, 2 µM of each dATP, dGTP, dTTP, dCTP, 1 µCi [$^{32}$P]dCTP (3000 Ci mmol$^{-1}$; Amersham, Aylesbury, UK) and 0.25 units of *Taq* DNA polymerase (Promega, Madison, WI, USA). PCR conditions were as follows: 30 cycles of denaturation at 94°C for 30 s, annealing at 50–60°C for 30 s, and elongation at 72°C for 30 s. After amplification, 2 µl of the reaction were mixed 1:1 with loading buffer (95% formamide, 20 mM EDTA, 0.05% bromophenol blue and 0.05% xylene cyanol), heat denatured and then electrophoresed through a 6% polyacrylamide/7 M urea gel. After electrophoresis, gels were vacuum dried and autoradiographed. A sample was scored positive for MI whenever somatic changes in the number of microsatellite repeat units or additional new alleles were observed in tumour DNA compared with normal DNA. LOH was defined as a > 50% reduction in intensity in one of the two alleles compared with those in normal tissue. To exclude technical aberrations or contamination, all the differences described between tumour and corresponding normal mucosa were confirmed by separate, independent amplification of different DNA preparations.

### Reverse transcriptase (RT)-PCR and *hMLH1* molecular analysis

RNA was extracted (Maestro et al. 1996) from matched tumours and corresponding normal mucosa obtained from 22 of the HNSCC patients analysed for MI, and cDNA was synthesized and examined for mutations in the entire coding region of the *hMLH1* gene. These cases had already been characterized for the presence of LOH at the 3p21.3 band and 15 showed LOH at this locus; moreover, eight of these patients were carriers of multiple respiratory tract malignancies.

Single-strand cDNA was synthesized by oligodeoxythymidylate priming from 1 µg of total RNA using 25 units of AMV RT (Promega) in a final volume of 20 µl, according to the manufacturer's instructions. After heat inactivation of the RT enzyme, the

© Cancer Research Campaign 1998

**Table 3** Primers used for *hMLH1* gene analysis

| Fragment size | Nucleotides | Primers |
|---|---|---|
| 2432 bp | 1–2396 | Sense 1:5′-GCATCTAGACGTTTCCTTGGC3′<br>Antisense 1:5′AGTATAAGTCTTAAGTGCTGCTACC3′ |
| 190 bp | 1–151 | Sense 1:5′-GCATCTAGACGTTTCCTTGGC3′<br>Antisense 2:5′-CAATCACTTGAATACTTG3′ |
| 290 bp | 103–393 | Sense 3:5′TGATTGAGAACTGTTTAG3′<br>Antisense 3:5′GAGTAACTTGCTCTGTAT3′ |
| 296 bp | 344–640 | Sense 4:5′TTACAAAGAAAACAGGCTG3′<br>Antisense 4:5′CCACGGTTGAGGCATTGG3′ |
| 195 bp | 592–787 | Sense 5:5′GAGAGACAGTAGCTGATG3′<br>Antisense 5:5′TGATGAAGAGTAAGAAGATGC3′ |
| 258 bp | 682–940 | Sense 6:5′TGATAGAAATTGGATGTGAGG3′<br>Antisense 6:5′CTTCATGCTTTGTGGGGT3′ |
| 289 bp | 894–1183 | Sense 7:5′CAGTCCCCAGAAATGTGGA3′<br>Antisense 7:5′CATCAAAGCTTCTGTTCCCG3′ |
| 284 bp | 1134–1418 | Sense 8:5′CTATGCCCACCAGATGGTT3′<br>Antisense 8:5′TGTCTCTTTCTGGGGTTG3′ |
| 264 bp | 1371–1635 | Sense 9:5′AATGTCAGAGAAGAGAGG3′<br>Antisense 9:5′GGTTTGATGCTGTGCCAA3′ |
| 269 bp | 1589–1858 | Sense 10:5′CGTGGGCTGTGTGAATCC3′<br>Antisense 10:5′CAGCCTTCTTCTTCAGAA3′ |
| 319 bp | 1806–2125 | Sense 11:5′TCCCAAAGAAGGACTTGCT3′<br>Antisense 11:5′GGAATGGAAGCCAGGCACT3′ |
| 315 bp | 2081–2396 | Sense 12:5′AGTCGACCCTCTCAGGCC3′<br>Antisense 1:5′AGTATAAGTCTTAAGTCTACC3′ |

product was diluted 1:5. A 2-μl aliquot of the diluted c-DNA was used directly for each PCR.

Molecular analysis of the *hMLH1* gene was performed by PCR-single-strand conformation polymorphism (PCR-SSCP), using a single strand of cDNA as template, and PCR-direct sequencing. In detail, the first PCR was carried out using a couple of sense 1 and antisense 1 primers. This amplificate was used as a template for subsequent PCR reactions using the other primers (see Table 3).

PCR-SSCP was performed following a two-step procedure. A first PCR was carried out in a 20-μl reaction using a 2-μl aliquot of cDNA, 20 pmol of sense 1 and antisense 1 primers, 200 μM of each dNTP, standard PCR-buffer (Promega), 0.1 μl (0.5 units) of *Taq* polymerase (Promega). Reaction mixtures were heated to 95°C for 3 min and then cycled 40 times with denaturation at 95°C for 1 min, annealing at 58°C for 1 min and elongation at 72°C for 2 min.

In the second step, 1 μl of the first PCR (diluted 1:1000) was reamplified in 10 μl of a reaction mixture containing 1 μCi of [$^{32}$P]dCTP (3000 Ci mmol$^{-1}$; Amersham), 2 μM of each dNTP and 10 pmol of sense and antisense primers (2–12), heated to 95°C for 3 min and then cycled 40 times with denaturation at 95°C, annealing at 55°C and elongation at 72°C, each for 30 s.

An aliquot of 2 μl of the reaction was mixed with 2 μl of formamide/EDTA/xylene cyanol bromophenol blue gel-loading buffer, heat-denatured and loaded into an ultrahigh resolution MDE gel (mutation detection enhancement, AT Biochem, Malvern, PA, USA), according to the manufacturer's instructions. Run conditions were 500 V overnight and 1000 V at room temperature with fan cooling for 5–7 h.

For PCR-direct sequencing, several independent 100-μl PCR-reactions were performed with 100 pmol of each primer and 5 μl of the first PCR (dilution 1:1000) as template, cycled 40 times, purified by 2% metaphor agarose (FMC) gel electrophoresis and extracted by the QIAEX Gel Extraction kit (QIAGEN, Chattsworth, CA, USA). DNA strand sequencing was performed

**Table 4** Cases with MI

| Cases | Loci with instability/loci analysed (locus) | |
|---|---|---|
| HN 12 | 1/20 | (D3S966) |
| HN 14[a] | 2/20 | (D3S1029–D3S1038) |
| HN 20[a] | 2/20 | (D3S647–DM) |
| HN 56 | 1/20 | (D3S1038) |
| HN 151 | 1/20 | (RB.20 VNTR) |

[a]Patients who developed MPTs.

**Figure 1** Microsatellite instability in squamous cell carcinomas of the head and neck. N, normal DNA; T, tumour DNA. The novel alleles in the tumour sample are indicated by the arrowhead. Top, cases HN 14 and HN 20 presented instability at two loci concurrently, D3S1029 and D3S1038 for HN 14, D3S647 and DM for HN 20. Bottom, the other cases (HN 56, HN 12 and HN 151) showed microsatellite instability at only one of the markers tested (D3S1038, D3S966 and RB.20VNTR) respectively by the dideoxy termination method using a Sequenase kit (USB, Cleveland, OH, USA). Reactions were loaded on 6% acrylamide/ 7 M urea TBE gel and run for 60 min at 45 W.

### RESULTS

The role of genetic instability in the development of HNSCC, and in particular in the propensity to multiple malignancies in the upper respiratory tract, was investigated in 67 HNSCC cases, 22 of which had MPT, by evaluating MI and LOH at 20 loci mapping to chromosomes 1p, 3p, 3q, 9p, 13q and 19p.

Genomic alterations compatible with MI and consisting of additional novel alleles in tumour DNA compared with its normal tissue counterpart were observed in only 5 out of 67 cases analysed (7%). No widespread microsatellite alterations were detected. Only two cases showed instability at two loci contemporarily (HN 14 and HN 20; Table 4, Figure 1). No correlation was found between MI and clinicopathological characteristics such as tumour stage, age at diagnosis and lifestyle habits (Table 1).

© Cancer Research Campaign 1998

**Figure 2** Representative examples of LOH in squamous cell carcinomas of the head and neck. N, normal DNA; T, tumour DNA. Cases HN 11 and HN 5 are patients with multiple tumours, case HN 12 is a patient with single tumour

No significant difference in the frequency of MI was detected in MPT cases compared with cases with single cancer. In fact, microsatellite alterations were observed in only 2 out of 22 (9%) patients with MPTs vs 3 out of 45 (7%) patients with a single cancer (Table 5).

As MI may give rise to apparent LOH as a consequence of the convergence in size of two originally different alleles, we also evaluated LOH at the same loci investigated for MI. No significant difference in the frequency of LOH at 1p, 3p, 9p, 13q and 19p was detected between MPT cases and cases with single tumour (Table 5). LOH at 1p was found in 3 of 19 (16%) cases with MPTs vs 16 of 41 (39%) cases with single tumour. The 3p chromosomal arm was lost in 17 of 22 (77%) cases with MPTs vs 28 of 40 (70%) cases with single tumour. The microsatellite marker D9S126 on chromosome 9p appeared to have LOH in 5 of 13 (38%) cases with MPTs vs 17 of 39 (44%) cases with single tumour. Loss of 13q was found in 15 of 21 (71%) cases with MPTs vs 30 of 44 (68%) other cases. The microsatellite marker DM on chromosome 19p showed LOH in 2 of 13 (15%) cases with MPTs vs 4 of 30 (13%) cases with single cancer. Some representative cases with LOH are reported in Figure 2. To evaluate the possible involvement of the mismatch repair *hMLH1* gene in MI, and as a potential preferential target of the deletions at 3p21, 22 cases, including the five cases showing MI, were analysed for *hMLH1* gene mutations by PCR-SSCP analysis and sequencing. Fifteen of these cases presented LOH at 3p21 region and eight developed MPTs. The analysis of the entire coding region of the *hMLH1* gene showed no somatic or germline *hMLH1* mutations.

### DISCUSSION

Genetic instability as a determinant of propensity to single and multiple malignancies of the upper respiratory tract was investigated in this study. MI was detected in only 5/67 (7%) cases analysed, indicating that defects in the mismatch repair mechanisms play a minor role in the development of HNSCC. The frequency of instability was not significantly increased in cases with MPTs in comparison with cases with single cancer. Microsatellite alterations were observed in only 2 out of 22 (9%) patients with MPTs vs 3 out of 45 (7%) patients with a single cancer. Similarly, no significant difference was observed in the frequency of LOH at the same loci between MPT and single primary tumour patients. A concordantly higher frequency of deletion was observed at 3p, 9p and 13q, whereas a relatively low frequency was detected at 1p and 19p both in patients with MPTs and in patients with single tumour. This indicates that genetic instability resulting in allele shift or gross deletions does not account for the propensity to develop single or multiple tumours in the upper aerodigestive tract. Accordingly, no somatic or germline mutations at the entire coding region of the *hMLH1* gene were detected in any of the cases analysed, also indicating that *hMLH1* is not the specific target of the deletion phenomena at 3p21 in HNSCC.

The overall low frequency of MI detected in our series is in agreement with previously published data reporting MI in 6/91 (7%) oral carcinomas (Ishwad et al, 1996), and in 5/56 (9%) HNSCCs (El-Naggar et al, 1995). A similar frequency has also been reported in oesophageal cancer (Muzeau et al, 1997), a tumour that shares histology and environmental risk factors with HNSCC. A slightly higher frequency was reported by El-Naggar et al (1996) and Field et al (1995) (15–30%). The different frequency may be related to features of the series as well as to number and type of loci tested. In fact, it has been reported that the frequency of instability may vary for different microsatellite markers (Wooster et al, 1994). Interestingly, in Field's study a role for MI was suggested in non-smoker HNSCC patients. Our series included only four non-smoker patients and, although four cases are insufficient to draw any conclusion, no MI was detected in these non-smoker patients. Furthermore, unlike El-Naggar et al (1996), who suggested that MI is involved in the late HNSCC progression stages, no differences in terms of tumour stage, age at diagnosis and lifestyle habits were observed between tumours with MI and those without it.

Recently, Horii et al (1994) reported an association between MI and development of MPT in the gastroenteric tract. Our results, in agreement with those reported by Shimada et al (1995) for oesophageal cancer, suggest that presumably different molecular mechanisms account for the tumour multiplicity phenomena in different anatomic sites. In particular, if genetic instability as a result of mismatch repair defects seems to be a major event in the genesis of sporadic and familial forms of gastroenteric tumours, defects in these mechanisms play a minor role in the tumorigenesis of the mucosa of the upper aerodigestive tract. Our data do not exclude that other forms of genetic instability may still be relevant

**Table 5** Alterations at microsatellite markers in cases with MPTs compared with cases with single tumour

| | No. of MI (%) | LOH at 1p (%)[a] | LOH at 3p (%)[a] | LOH at 9p (%)[a] | LOH at 13q (%)[a] | LOH at 19p (%)[a] |
|---|---|---|---|---|---|---|
| Cases with MPTs | 2/22 (9) | 3/19 (16) | 17/22 (77) | 5/13 (38) | 15/21 (71) | 2/13 (15) |
| Cases with single tumour | 3/45 (7) | 16/41 (39) | 28/40 (70) | 17/39 (44) | 30/44 (68) | 4/30 (13) |

No. of cases with MI per cases analysed; [a]Cases with LOH per informative cases.

© Cancer Research Campaign 1998

in the genesis of this type of tumour. In particular, it has been suggested that other mechanisms controlling the integrity of the genome but not related to the mismatch repair are likely to be disrupted in the fraction of HNPCC patients that do not show MI (Liu et al, 1996; Moslein et al, 1996). It will be interesting to investigate the role of these mechanisms, once identified, in the development of head and neck carcinomas and in the phenomena of field cancerization of the upper aerodigestive tract.

### ACKNOWLEDGEMENTS

This work was supported in part by a grant from the Italian Association for Cancer Research. S Piccinin is the recipient of a fellowship from the Italian Foundation for Cancer Research. The authors thank Ms P Tonel for help with the manuscript.

### REFERENCES

Benatti P, Sassatelli R, Roncucci L, Pedroni M, Fante R, Di Gregorio C, Losi L, Gelmini R and Ponz De Leon M (1993) Tumor spectrum in hereditary non-polyposis colorectal cancer (HNPCC) and in families with 'suspect HNPCC': a population-based study in northern Italy. *Int J Cancer* **54**: 371–377

Bonadonna G and Robustelli della Cuna G (1991) *Manuale di Oncologia Medica*, pp. 589–590. Masson: Milan

Gloos J, Boubewijn JMB, Steen I, Cooper MP, De Vries N, Nauta JJP and Snow GB (1994) Increased mutagen sensitivity in head-and-neck squamous cell carcinoma patients, particularly those with multiple primary tumours. *Int J Cancer* **56**: 816–819

Copper MP, Jovanovic A, Nauta JJP, Braakhuis BJM, de Vries N, van der Waal I and Snow GB (1995) Role of genetic factors in the etiology of squamous cell carcinoma of the head and neck. *Arch Otolaryngol Head Neck Surg* **121**: 157–160

El-Naggar AK, Hurr K, Huff V, Luna MA, Goepfert H, Batsaki JG (1995) Allelic loss and replication error at microsatellite loci on chromosome 11p in head and neck squamous carcinoma: association with aggressive biological features. *Clin Cancer Res* **2**: 903–907

El-Naggar AK, Hurr K, Huff V, Clayman GL, Luna MA and Batsakis JG (1996) Microsatellite instability in preinvasive and invasive head and neck squamous carcinoma. *Am J Pathol* **148**: 2067–2072

Engelstein M, Hudson TJ, Lane JM, Lee MK, Leverone B, Landes GM, Peltonen L, Weber JL and Dracopoli NC (1993) A PCR-based linkage map of human chromosome 1. *Genomics* **15**: 251–258

Field JK, Kiaris H, Howard P, Vaughan ED, Spandidos DA and Jones AS (1995) Microsatellite instability in squamous cell carcinoma of the head and neck. *Br J Cancer* **71**: 1065–1069

Fountain JW, Hudson TJ, Engelstein M, Housman DE and Dracopoli NC (1993) Dinucleotide repeat polymorphism at the D9S126 locus (9p21). *Human Mol Genet* **2**: 823

Franceschi S, Talamini R, Barra S, Baron AE, Bidoli E, Serraino D and La Vecchia C (1990) Smoking and drinking in relation to cancers of the oral cavity, pharynx, larynx, and esophagus in Northern Italy. *Cancer Res* **50**: 6502–6507

Franceschi S, Bidoli E, Baron AE, Barra S, Talamini R, Serraino D and La Vecchia C (1991) Nutrition and cancer of the oral cavity and pharynx in north-east Italy. *Int J Cancer* **47**: 20–25

Granqvist M, Xiang K, Seino M, Fukumoto H and Bell GI (1991) Dinucleotide repeat polymorphism in human GLUT2/liver facilitative glucose transporter gene on chromosome 3. *Nucleic Acid Res* **19**: 4791

Hara H, Ozeki S, Shiratsuchi Y, Tashiro H and Jingu K (1988) Familial occurrence of oral cancer: report of cases. *J Oral Maxillofac Surg* **46**: 1098–1102

Horii A, Han H-J, Shimada M, Yanagisawa A, Kato Y, Yasui W, Tahara E and Nakamura Y (1994) Frequent replication errors at microsatellite loci in tumors of patients with multiple primary cancers. *Cancer Res* **54**: 3373–3375

Ishwad CS, Ferrell R, Rossie KM, Appel BN, Johnson JT, Myers EN, Law JC, Srivastava S and Gollin SM (1996) Microsatellite instability in oral cancer. *Int J Cancer* **64**: 332–335

Liu B, Parsons R, Papadopoulos N, Nicolaides NC, Lynch HT, Watson P, Jass JR, Dunlop M, Wyllie A, Peltomäki P, de la Chapelle A, Hamilton SR, Vogelstein B and Kinzler KW (1996) Analysis of mismatch repair genes in hereditary non-polyposis colorectal cancer patients. *Nature Med* **2**: 169–174

Lynch HT, Kriegler M, Christiansen TA, Smyrk T, Lynch JF and Watson P (1988) Laryngeal carcinoma in a Lynch syndrome II kindred. *Cancer* **62**: 1007–1013

Lynch HT, Smyrk TC, Watson P, Lanspa SJ, Lynch JF, Lynch PM, Cavalieri RJ and Boland CR (1993) Genetics, natural history, tumor spectrum, and pathology of hereditary nonpolyposis colorectal cancer: an updated review. *Gastroenterology* **104**: 1535–1549

Maestro R, Gasparotto D, Vukosavljevic T, Sulfaro S, Barzan L and Boiocchi M (1993) Three discrete regions of deletion at 3p in head and neck cancers. *Cancer Res* **53**: 5775–5779

Maestro R, Piccinin S, Doglioni C, Gasparotto D, Vukosavljevic T, Sulfaro S, Barzan L and Boiocchi M (1996) Chromosome 13q deletion mapping in head and neck squamous cell carcinomas: identification of two distinct regions of preferential loss. *Cancer Res* **56**: 1146–1150

Mäkelä TP, Hellstein E, Vesa J, Alitano K and Peltonen L (1992) An Alu variable polyA repeat polymorphism upstream of L-myc at lp32. *Human Mol Genet* **1**: 217

Modrich P (1994) Mismatch repair, genetic stability, and cancer. *Science* **266**: 1959–1960

Moslein G, Tester DJ, Lindor NM, Honchel R, Cunningham JM, French AJ, Halling KC, Schwab M, Goretzki P and Thibodeau SN (1996) Microsatellite instability and mutation analysis of hMSH2 and hMLH1 in patients with sporadic, familial and hereditary colorectal cancer. *Human Mol Gen* **5**: 1245–1252

Muzeau F, Fléjou J-F, Belghiti J, Thomas G and Hamelin R (1997) Infrequent microsatellite instability in oesophageal cancers. *Br J Cancer* **75**: 1336–1339

Richards RI and Sutherland GR (1994) Simple repeat DNA is not replicated simply. *Nature Gen* **6**: 114–116

Schantz SP, Spitz MR and Hsu TC (1990) Mutagen sensitivity in patients with head and neck cancers: risk of multiple primary malignancies. *J Natl Cancer Inst* **82**: 1773–1775

Schwartz LH, Ozsahin M, Zhang GN, Touboul E, De-vataire F, Andolenko P, Lacan-Saint-Guily J, Lougier A and Schlienger M (1994) Synchronous and metachronous head and neck carcinomas. *Cancer* **74**: 1933–1938

Shimada M, Horii A, Sasaki S, Yanagisawa A, Kato Y, Yamashita K, Okagawa K, Yamasaki K, Ishiguro S, Inoue M, Shiosaki H and Nakamura Y (1995) Infrequent replications errors at microsatellite loci in tumors of patients with multiple primary cancers of the esophagus and various other tissues. *Br J Cancer* **86**: 511–515

Slaughter TP, Southwich HW and Smejkel W (1953) Field cancerization in oral stratified epithelium. *Cancer* **6**: 963–968

Tashiro H, Abe K and Tanioka H (1988) Familial occurrence of cancer of the mouth: report of cases. *J Oral Maxillofac Surg* **44**: 322–323

Wooster R, Cleton-Jansen AM, Collins N, Mangion J, Cornelis RS, Cooper CS, Gusterson BA, Ponder BAJ, von Deimling A, Wiestler OD, Cornelisse CJ, Devilee P and Stratton MR (1994) Instability of short tandem repeats (microsatellites) in human cancers. *Nature Genet* **6**: 152–156

© Cancer Research Campaign 1998